# Determinants of new onset cardiometabolic risk among normal weight children

**DOI:** 10.1038/s41366-019-0483-0

**Published:** 2019-11-25

**Authors:** Andraea Van Hulst, Marina Ybarra, Marie-Eve Mathieu, Andrea Benedetti, Gilles Paradis, Mélanie Henderson

**Affiliations:** 1 Ingram School of Nursing, McGill University, Montreal, QC Canada; 2 CHU Sainte-Justine Research Centre, Montreal, QC Canada; 3 Institut Armand Frappier, Laval, QC Canada; 4 Department of Kinesiology, Université de Montréal, Montreal, QC Canada; 5 Department of Epidemiology Biostatistics and Occupational Health, McGill University, Montreal, QC Canada; 6 Department of Medicine, McGill University, Montreal, QC Canada; 7 Department of Pediatrics, Université de Montréal, Montreal, QC Canada

**Keywords:** Metabolism, Endocrinology

## Abstract

**Objective:**

To identify determinants for the development of “normal weight metabolically unhealthy” (NWMU) profiles among previously metabolically healthy normal weight children.

**Methods:**

The QUALITY cohort comprises youth 8–10 years of age with a parental history of obesity (*n* = 630). Of these, normal weight children with no metabolic risk factors were identified and followed up 2 years later (*n* = 193). Children were classified as NWMU if they remained normal weight but developed at least one cardiometabolic risk factor. They were classified as normal weight metabolically healthy otherwise. Multivariable logistic regression models were used to identify whether adiposity (anthropometrics and DXA), lifestyle habits (physical activity, screen time, vegetables, and fruit- and sugar-sweetened beverages intake), fitness, and family history of cardiometabolic disease were associated with new onset NWMU.

**Results:**

Of the 193 normal weight and metabolically healthy children at baseline, 45 (23%) became NWMU 2 years later (i.e., 48% had elevated HDL cholesterol, 13% had elevated triglycerides, and 4% had impaired fasting glucose). Changes in adiposity between baseline and follow-up were associated with an increased risk of NWMU for all adiposity measures examined (e.g., for ∆zBMI OR = 3.95; 95% CI: 1.76, 8.83). Similarly, a 2-year change in screen time was associated with incident NWMU status (OR = 1.24; 95% CI 1.04, 1.49).

**Conclusions:**

Children who increase their adiposity levels as they enter puberty, despite remaining normal weight, are at risk of developing cardiometabolic risk factors. Studies examining long-term consequences of NWMU profiles in pediatrics are needed to determine whether changes in screening practice are warranted.

## Introduction

The clustering of cardiometabolic risk factors [1–3] is strongly correlated with overweight and obesity in children and adolescents [4]. One unique subgroup of individuals comprises those who, despite having a normal weight based on body mass index (BMI) definitions, present a clustering of cardiometabolic risk factors. The normal weight but metabolically unhealthy (NWMU) phenotype was first clinically described 30 years ago [5]. According to a recent review, the prevalence of this phenotype in adults is nearly 50% in some populations [6]. Determinants and consequences of the NWMU phenotype have been examined primarily in adults [6–10]. Normal weight adults who present with a clustering of cardiometabolic risk factors appear to have a higher risk of mortality compared to healthy lean individuals without risk factor clustering, and possibly even compared to individuals with overweight/obesity [6]. A better understanding of the determinants of this phenotype is needed to inform prevention strategies.

Little is known regarding the NWMU phenotype in children and youth, yet this may be a particularly vulnerable subgroup as their cardiometabolic risk may remain undetected for long periods of time given their normal weight status. Although family history of type 2 diabetes and hypertension, higher fat mass and higher birth-weight have been associated with the NWMU phenotype in children [11, 12], determinants of incident, or new onset, NWMU status are unknown. A better understanding of these determinants will help clinicians identify high risk normal weight children who might benefit from cardiometabolic screening and early prevention. Therefore, we aimed to identify determinants for the development of new onset NWMU status among previously normal weight and metabolically healthy children as they enter puberty. We hypothesized that higher baseline adiposity and greater 2-year increases in adiposity, as well as poorer baseline lifestyle habits and 2-year deteriorations in lifestyle habits, are associated with an increased risk for new onset NWMU.

## Patients and methods

**Fig. 1:**
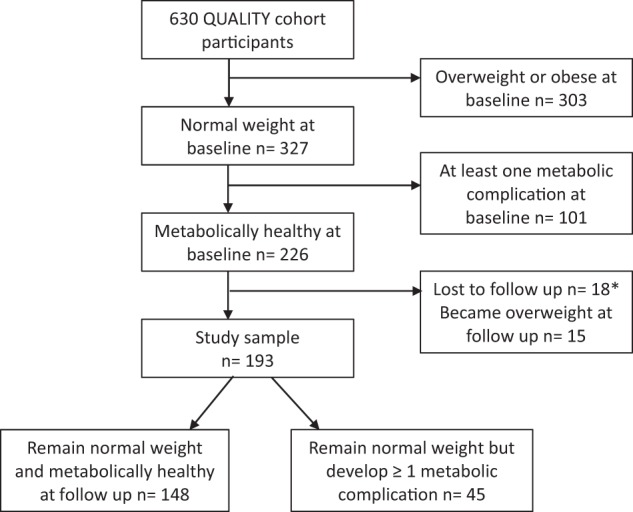
Participant flow diagram. *No statistically significant differences were found for baseline characteristics between participants included in the current analysis (*n* = 193) versus those lost to follow up (*n* = 18)

Participants were drawn from the QUALITY (QUebec Adipose and Lifestyle InvesTigation in Youth) cohort, an ongoing longitudinal investigation of the natural history of obesity and cardiovascular disease risk factors in youth. Children were recruited through elementary schools located in three urban centers in Quebec (Canada) using recruitment flyers. Participants were required to be Caucasian, aged 8–10 years at recruitment, and both biological parents had to be available to participate in baseline data collection, with at least one of them having obesity based on self-reported weight, height and waist circumference. At baseline, data were collected on 630 families (2005–2008). A similar assessment was conducted 2 years later, when children were aged 10–12 years (*n* = 564). For the current analysis, we used a sub-sample of QUALITY participants (*n* = 193), namely those who were normal weight and metabolically healthy at baseline and who remained normal weight at follow-up (Fig. 1). Written informed consent and assent were obtained from parents and children, respectively. The Ethics Review Boards of the CHU Sainte-Justine and the Quebec Heart and Lung Institute approved the study. A detailed description of the study design, standardized data collection methods and quality assurance procedures is available elsewhere [13].

### Measurements

#### Primary outcome variable

Participants were classified as NWMU if they had a normal weight (BMI < 85th percentile for age and sex) and had at least one of the following cardiometabolic risk factors: triglycerides > 1.24 mmol/L, fasting glucose > 6.1 mmol/L, high density lipoprotein (HDL) cholesterol < 1.03 mmol/L, systolic and/or diastolic blood pressure (BP) > 90th percentile for age, sex, and height, or waist circumference > 90th percentile for age and sex based on cutoffs proposed by Cook et al. [14]. We have previously reported that, whether considered individually or as a cluster, cardiometabolic risk factors as defined by Cook et al. predict lower insulin sensitivity among children entering puberty suggesting that having even a single cardiometabolic risk factor may be considered “unhealthy” [15]. Participants with a normal weight and no metabolic risk factors were classified as normal weight and metabolically healthy.

Variables used to define the cardiometabolic risk among normal weight children were obtained at baseline (8–10 years) and follow-up (10–12 years). Height, weight, waist circumference and BP were measured according to standardized protocols [13]. Age- and sex-specific BMI percentiles and *z*-scores were calculated according to WHO reference values [16]. Waist circumference was measured using a standard measuring tape placed at the mid-point between the last floating rib and the iliac crest at the end of a normal expiration; age- and sex-specific waist circumference percentiles were computed [17]. BP was measured on the right arm with the child in a sitting position, at rest for at least five minutes, using an oscillometric instrument. Five measures were obtained at one-minute intervals and the average of the last three was used. Age-, sex-, and height-specific percentiles for systolic and diastolic BP were computed using reference data from the National High Blood Pressure Education Program [18]. Blood samples were obtained by venipuncture after a 12-h overnight fast at both baseline and follow-up. Blood samples were centrifuged, aliquoted, and stored at −80 °C until analyzed. Fasting glucose, HDL cholesterol and triglycerides were measured on a Synchron LX^®^20 analyzer, with Beckman Instruments reagents, by the Department of Clinical Biochemistry at CHU Sainte-Justine, according to the recommendations of the International Federation of Clinical Chemistry.

#### Measurement of exposure variables

In addition to BMI *z*-scores (zBMI) described previously, waist-to-height ratio (WHtR) was computed as the ratio of waist circumference in cm by height in cm. Body composition was measured by dual-energy X-ray absorptiometry (DXA), including total body, android, and gynoid fat masses. The android region was defined using the pelvis cut as lower boundary and an upper boundary above the pelvis cut by 20% of the distance between the pelvis and neck cuts. The gynoid region was defined using upper boundary below the pelvis cut line by 1.5 times the android space and gynoid space was equal to 2 times the android space [19]. The ratio of android to gynoid fat (A/G fat ratio) was computed. The percentage of total body fat mass was computed as total fat mass/total body mass × 100. All adiposity measures were obtained at baseline and follow-up.

Physical activity was assessed at baseline and follow-up during a 7-day period using an Actigraph LS 7164 activity monitor (Actigraph, Pensacola, FL). Accelerometry data were downloaded as 1-min epochs and underwent standardized quality control and data reduction procedures [20]; participants with a minimum of 4 days and a minimum of 10 h of wear time per day were retained [21]. Non-wear time was defined as any period of 60 min or more of 0 counts, accepting 1–2 consecutive minutes where count values were higher than 0 and lower than or equal to 100 [22]. Moderate-to-vigorous physical activity (MVPA) was computed by adding the total minutes spent daily in moderate (≥2296 counts per minute) and in vigorous physical activity (≥4012 counts per minute) [23] and averaging over the total number of valid days of wear [24].

Cardiorespiratory fitness was estimated using peak oxygen consumption (VO_2 peak_) during an adapted standard incremental exercise test on an electromagnetic bicycle to volitional exhaustion with indirect calorimetry measurements throughout the test [13]. VO_2 peak_ was considered as a true maximum value if a respiratory exchange ratio (CO_2_ production to O_2_ consumption) > 1.0 or/and a heart rate > 185 beats/min was attained [25]. VO_2 peak_ was expressed as a function of lean body mass.

Screen time was assessed by interviewer-administered questionnaire at baseline and follow-up, documenting daily hours of television viewing and leisure computer or video game use on a typical week-day and weekend day; average daily hours of screen time was computed.

Children’s dietary intake was measured at baseline only using mean values of three 24-h diet recalls conducted by trained dieticians on nonconsecutive days including 1 weekend day [26]. Diet recall interviews were done by telephone, within a 4-week period following the baseline visit, with the child and the parent who prepared the meals.

Children reporting unreasonable intakes were questioned further to ascertain their intake. Prior to the first recall, participants were given a small disposable kit containing food portion models (e.g., graduated cup and bowl) as well as a short training session on how to report portion sizes during telephone interviews. Reported foods were entered into CANDAT (London, ON, Canada) and converted to nutrients using the 2007 Canadian Nutrient File. Outliers in the analysis of the distribution of each nutrient were examined; records of intakes with very high or low values of a nutrient were examined for any data entry mistakes. Total energy intake was measured in kilocalories. Daily servings of vegetables and fruit were based on portion sizes from the 2007 Canada Food Guide and include 100% fruits and vegetables juices. Sugar-sweetened beverage intake, including soft drinks and other sugary drinks, was measured as the average number of 100 mL portions per day.

Pubertal development stage was assessed by trained nurses using the 5-stage Tanner scales [27, 28], and was dichotomized as prepubertal (Tanner 1) vs puberty initiated (Tanner > 1) at baseline and follow-up.

Lastly, at baseline both biological parents completed a self-reported questionnaire on their history of physician-diagnosed high blood pressure, dyslipidemia and diabetes. Parents also underwent standardized anthropometrics and fasting blood tests (as described for children); metabolic syndrome among parents was determined using the NCEP-Adult Treatment Panel III criteria [29] and classified > 1 vs 0 parents with the metabolic syndrome.

### Analyses

Means, medians, and proportions of participants’ baseline characteristics were compared between normal weight children who remained metabolically healthy and those who developed new onset NWMU using independent samples *t*-tests, Wilcoxon tests or chi-squared tests, respectively. We also compared group differences for 2-year changes in adiposity, lifestyle habits, and fitness.

Multivariable logistic regressions were used to estimate the risk of new onset NWMU at follow-up (10–12 years) associated with each baseline exposure variable in separate models. We also examined whether 2-year changes in adiposity, physical activity, screen time, and fitness, between baseline and follow-up, accounting for baseline values, were associated with new onset NWMU. All models were adjusted for the participants’ sex, age, and Tanner stage at follow-up; models including dietary intake were additionally adjusted for total kilocalorie intake, and models examining the effect of 2-year changes in adiposity, lifestyle habits and fitness were additionally adjusted for corresponding baseline variables. To ease the interpretation of odds ratios (OR) associated with WHtR and A/G fat ratio, variables were standardized by subtracting values by the sample’s mean and dividing by the standard deviation (SD). Thus, ORs correspond to the risk of developing new onset NWMU for 1 SD increase in WHtR and A/G fat ratio from baseline to follow-up. Lastly, given that physical activity data were missing for 10% of cases at baseline and 27% of cases at follow-up, and VO_2_ peak data were missing in 6% of cases at baseline and 9% of cases at follow-up, we repeated logistic regressions on 20 imputed data sets created using multiple imputation with the fully conditional specification. These results are presented as sensitivity analyses. Statistical analyses were performed using the SAS version 9.4 (Cary, North Carolina).

## Results

**Fig. 2:**
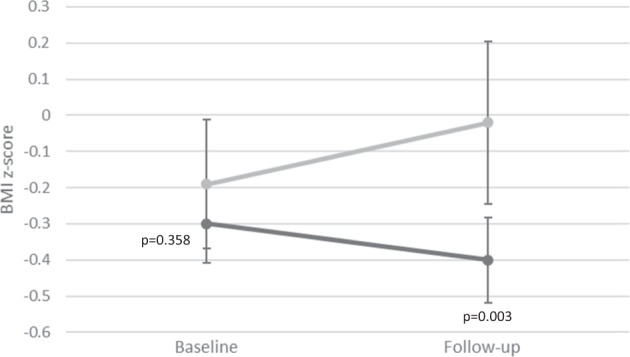
Changes in BMI *z*-score between baseline and follow-up among children who remain normal weight and metabolically healthy and those who develop incident NWMU. Dark gray (or black): remain metabolically healthy, Light gray: new onset NWMU

Among the metabolically healthy participants at baseline, 15 were no longer eligible because they developed overweight at follow-up, and 18 were lost to follow-up (Fig. 1). Baseline characteristics did not differ between the children lost to follow-up and those included in this study (*n* = 193). Of the 193 children who were normal weight and metabolically healthy at baseline (8–10 years), 45 (23%) developed the NWMU phenotype. Of those, 43 had developed 1 and 2 had developed 2 cardiometabolic risk factor. The most common risk factor was low HDL cholesterol followed by high triglycerides (Table 1). The 45 incident cases of NWMU were more likely to have initiated puberty at follow-up and had, on average, a higher percent body fat at baseline compared to children who remained healthy (Table 2). No other differences in baseline adiposity, lifestyle habits or fitness were found. Over the 2 years of follow-up, those who developed the NWMU phenotype experienced a larger increase in zBMI (Fig. 2). For example, 64% of those who developed the NWMU phenotype compared to 38% of those who remained healthy experienced any increase in zBMI over the 2 years of follow-up (data not shown). Maternal history of hypertension was found to be more prevalent among incident cases of NWMU than among those who remained healthy.

**Table 1:** Prevalence of risk factors defining participants as metabolically unhealthy at 10–12 years (*n* = 45)

Risk factors	% (*n*)
HDL cholesterol ≤ 1.03 mmol/L	46.7 (21)
Triglycerides ≥ 1.24 mmol/L	13.3 (6)
Fasting glucose ≥ 6.1 mmol/L	4.4 (2)
SBP > 90th percentile for age, sex, and height	0
DBP > 90th percentile for age, sex, and height	0
Waist circumference > 90th percentile for age and sex	0
Number of cardiometabolic risk factors	
1	95.6 (43)
2	4.4 (2)
3 or more	0

*DBP* diastolic blood pressure, *HDL* high density lipoprotein, *SBP* systolic blood pressure

**Table 2:** Description of participants by metabolic status at follow-up among 193 previously metabolically healthy normal weight children from the QUALITY cohort

	Metabolically unhealthy (*n* = 45)	Metabolically healthy (*n* = 148)	*p* value*
	Mean ± SD or % or median (Q1–Q3)		
Boys	48.9	60.1	0.181
Tanner stage > 1 at baseline	20.0	10.1	0.079
Tanner stage > 1 at follow-up	73.3	53.7	0.020
Age at baseline, years	9.7 ± 0.9	9.5 ± 0.9	0.166
Age at follow-up, years	11.7 ± 0.9	11.6 ± 1.0	0.323
Adiposity at baseline			
zBMI	−0.2 ± 0.6	−0.3 ± 0.7	0.358
Percent fat mass	18.2 ± 5.9	15.8 ± 5.2	0.010
WHtR	0.43 ± 0.020	0.42 ± 0.021	0.284
A/G fat ratio	0.23 ± 0.060	0.21 ± 0.045	0.088
Lifestyle habits and fitness at baseline			
MVPA, min/day, median (IQR)	46.4 (30.6 to 66.1)	59.6 (37.9 to 77.0)	0.109
Screen time, h/day	2.6 ± 1.9	2.3 ± 1.7	0.297
Total energy intake, kcal	1712 ± 392	1707 ± 364	0.928
Vegetables and fruits, portions/day	4.1 ± 2.1	4.5 ± 2.1	0.249
Sugar-sweetened beverage, mL/day, median (IQR)	83.2 (0 to 238.1)	66.7 (0 to 166.3)	0.384
VO_2_ peak, mL/Kg LBM/min	58.3 (53.1 to 62.6)	57.9 (54.5 to 62.1)	0.824
2-year change in adiposity, lifestyle and fitness			
∆ zBMI	0.2 ± 0.5	−0.11 ± 0.46	<0.001
∆ percent fat mass	2.8 ± 5.3	2.0 ± 3.7	0.367
∆ WHtR	−0.0038 ± 0.021	−0.0095 ± 0.018	0.068
∆ A/G fat ratio	0.023 ± 0.039	0.0072 ± 0.041	0.030
∆ MVPA, min/day, median (IQR)	−4.0 (−19.7 to 10.0)	−6.3 (−24.3 to 5.0)	0.560
∆ screen time, h/day	1.3 ± 2.2	0.6 ± 1.9	0.052
∆ VO_2_ peak	0.3 (−2.2 to 4.7)	1.9 (−1.7 to 5.8)	0.147
Family history of cardiometabolic disease			
≥1 parent with metabolic syndrome	71.1	60.1	0.183
History of maternal hypertension	15.6	6.1	0.044
History of paternal hypertension	33.3	23.7	0.194
History of maternal dyslipidemia	62.2	69.6	0.354
History of paternal dyslipidemia	97.8	93.2	0.251

*A/G fat ratio* android to gynoid fat ratio, *IQR* interquartile range, *LBM* lean body mass, *MVPA* moderate-to-vigorous physical activity, *WHtR* waist-to-height ratio, *zBMI* body mass index *z*-score

*p values are for Chi-square tests when comparing proportions, independent sample *t*-tests when comparing means, and Wilcoxon tests when comparing medians by metabolic status among normal weight children

Results from multivariable logistic regressions are shown in Table 3. In this sample of normal weight children, although baseline adiposity was not a predictor of new onset NWMU, 2-year change in adiposity was associated with an increased risk of incident NWMU for the 4 adiposity measures considered. For example, a 1 SD increase in zBMI over 2 years resulted in a fourfold increased risk of incident NWMU (OR: 3.95, 95% CI: 1.76–8.83). Similarly, a 1 SD increase in WHtR over 2 years increased the risk of NWMU by 60% (OR: 1.59, 95% CI: 1.09–2.32). In terms of lifestyle behaviors, trends towards protective associations were found for baseline MVPA and intake of vegetables and fruits but these did not reach statistical significance. Although baseline screen time was not associated with incident NWMU, an association was observed for 2-year changes in screen time: every additional increase in 1 h/day of screen time from baseline to follow-up was associated with a 24% greater risk of NWMU (OR: 1.24, 95% CI: 1.04–1.49). Family history of cardiometabolic disease was not associated with the development of new onset NWMU. Findings were similar when using imputed data.

**Table 3:** Associations between exposures and the likelihood of developing the incident NWMU profile among 193 previously metabolically healthy normal weight children from the QUALITY cohort

	Odds ratio (95% CI)	
	Case complete	Imputed data
Adiposity at baseline		
zBMI	1.29 (0.74, 2.23)	1.28 (0.74, 2.20)
Percent fat mass (%)	1.06 (0.99, 1.14)	1.06 (0.99, 1.14)
WHtR (1 SD)	1.28 (0.90, 1.83)	1.28 (0.90, 1.83)
A/G fat ratio (1 SD)	1.33 (0.95, 1.87)	1.31 (0.93, 1.83)
Lifestyle habits and fitness at baseline		
MVPA (10 min/day)	0.86 (0.73, 1.02)	0.88 (0.75, 1.03)
Screen time (h/day)	1.13 (0.93, 1.37)	1.12 (0.92, 1.36)
Fruits and vegetables (portions/day)^a^	0.88 (0.73, 1.05)	0.88 (0.73, 1.05)
Sugar-sweetened beverages (100 mL/day)^a^	1.16 (0.92, 1.48)	1.17 (0.92, 1.48)
VO_2_ peak (mL/kg LBM/min)	1.01 (0.95, 1.08)	1.01 (0.96, 1.08)
2-year changes in adiposity, lifestyle habits, and fitness^b^		
∆ zBMI	3.95 (1.76, 8.83)	3.98 (1.78, 8.92)
∆ percent fat mass (%)	1.10 (1.00, 1.20)	1.09 (1.00, 1.19)
∆ WHtR (1 SD)	1.59 (1.09, 2.32)	1.59 (1.09, 2.31)
∆ A/G fat ratio (1 SD)	1.83 (1.23, 2.73)	1.68 (1.15, 2.47)
∆ MVPA (min/day)	0.98 (0.96, 1.01)	0.98 (0.96, 1.01)
∆ screen time (h/day)	1.24 (1.04, 1.49)	1.24 (1.04, 1.49)
∆ VO2 peak (mL/kg LBM/min)	1.00 (0.93, 1.07)	0.99 (0.93, 1.06)
Family history of cardiometabolic disease at baseline		
≥1 parent with metabolic syndrome (vs none)	1.54 (0.73, 3.24)	1.57 (0.75, 3.29)
Family history of CVD	1.19 (0.57, 2.49)	1.18 (0.56, 2.46)
History of hypertension in mother	2.71 (0.91, 8.05)	2.72 (0.92, 8.08)
History of hypertension in father	1.44 (0.69, 3.04)	1.46 (0.69, 3.07)
History of dyslipidemia in mother	0.77 (0.38, 1.54)	0.76 (0.37, 1.54)
History of dyslipidemia in father	3.49 (0.42, 28.63)	3.47 (0.42, 28.52)

All models are adjusted for sex, age and Tanner stage at follow-up

*A/G fat ratio* android to gynoid fat ratio, *LBM* lean body mass, *MVPA* moderate-to-vigorous physical activity, *WHtR* waist-to-height ratio, *zBMI* body mass index *z*-score

^a^Indicates that models are additionally adjusted for total kilocalorie intake

^b^Indicates that models are additionally adjusted for corresponding baseline measure of adiposity (i.e., zBMI, % fat mass, WHR, A/G ratio) or lifestyle habits (i.e., MVPA, screen time) so as to estimate associations for 2-year changes in these exposure variables, accounting for baseline level

## Discussion

Among children with a parental history of obesity, we observed that close to one in four children who were normal weight and metabolically healthy at age 8–10 years developed at least one cardiometabolic risk factor 2 years later, even though they remained normal weight. Increases in adiposity as measured by zBMI, percent fat mass, WHtR, and A/G ratio, despite remaining normal weight, and increasing use of screen-based activities over 2 years from childhood to early adolescence were identified as determinants for the new onset NWMU phenotype.

Following adjustment for age, sex, and pubertal development, baseline percentage of body fat was only marginally associated with an increased risk of new onset NWMU. Others have reported that normal weight children who present with cardiometabolic risk factors have higher adiposity levels [30]. Similarly, a longitudinal study of girls followed into young adulthood reported that BMI-defined normal weight girls who had a higher percent body fat mass were more likely to develop cardiometabolic risk factors compared to those with a normal weight and a lower percent body fat mass [31]. In adults, it was also shown that NWMU women have a higher percentage of body fat [7]. However, most studies have examined adiposity in prevalent cases whereas we studied incident cases of NWMU, which likely have different determinants. Our findings are in-line with a recently published prospective study in adults showing that adiposity irrespective of BMI-defined weight status increases cardiometabolic disease morbidity [32].

Although baseline zBMI was not associated with new onset NWMU in this sample of normal weight children, we found a 2-year increase in zBMI from childhood to early adolescence to be a strong determinant of NWMU, even after accounting for pubertal development, sex and age. On average, children who developed the NWMU phenotype increased their zBMI by 0.2 SD while those who remained healthy decreased their zBMI by 0.1 SD. An increase in adiposity is expected in the age range examined, particularly among girls entering puberty. Nonetheless, all four measures of adiposity predicted incident NWMU in this cohort, independent of confounding by pubertal development, sex, and age. Among youth with obesity but who are metabolically healthy, it has been shown that excessive weight gain (>0.12 SD increase in zBMI over 2 years) is associated with the development of the metabolic syndrome [33]. Other studies have found rapid weight gain early in childhood to be associated with cardiometabolic risk factors in later childhood [34, 35]. These findings suggest that a child presenting with an increase in zBMI over time may need to be monitored for the development of cardiometabolic risk factors, even when the BMI values remain below the age and sex-adjusted cutoff for overweight.

The NWMU phenotype has also been characterized by greater visceral adipose tissue and ectopic fat deposition [6, 30], which are associated with inflammation and may be key in the pathogenesis of cardiometabolic risk factors in normal weight children [36]. We observed that a 2-year increase in WHtR, an indicator of abdominal obesity [37], is associated with incident NWMU; findings were similar when using a DXA-derived measure of abdominal obesity. We used a waist circumference > 90^th^ percentile for age and sex as a cardiometabolic risk factor defining NWMU. However, in this sample of normal weight children, none met this criteria meaning that all participants included in this analysis had a normal waist circumference at baseline and follow-up. Our findings suggest that even within the normal range of waist circumference, 2-year increases in abdominal adiposity as measured by WHtR and A/G fat ratio are determinants of incident NWMU. These findings highlight the importance of measuring and monitoring waist circumference in pediatric clinics, even among normal weight children.

Although we found limited evidence for associations between lifestyle habits or fitness and the risk of developing the NWMU phenotype, our findings point towards the detrimental impact of increasing screen time from childhood to early adolescence on cardiometabolic health, even among normal weight children [38]. Moreover, baseline physical activity, but not 2-year changes in physical activity, was marginally protective against the risk of developing the NWMU phenotype. It may be that much higher levels of physical activity are required to successfully prevent the development of metabolic complications than the levels engaged in by QUALTY study participants. Similarly, daily portions of vegetables and fruits were negatively associated with new onset NWMU but did not reach statistical significance. Few studies have investigated lifestyle habits in relation to the NWMU phenotype in children and youth. A recent study in Asian adults showed improvements in the metabolic profile of NWMU participants after a diet-induced 5% weight loss [39]. Another study in adult women reported that, compared to a metabolically healthy group, NWMU women had lower physical activity levels and lower energy expenditure [40]. Given the small sample size of our study, we were not sufficiently powered to detect potential small effects of lifestyle habits. Nevertheless, this study points to a deleterious effect of increasing screen time from childhood to early adolescence and the possible protective effect of physical activity and vegetable and fruit intake in childhood on subsequent risk of new onset NWMU in early adolescence.

In this study, family history of cardiometabolic disease was not a determinant of new onset NWMU. Other studies have reported that a family history of diabetes was associated with the clustering of cardiometabolic risk factors, independently of weight status [11, 41], as was family history of hypertension [41, 42]. The wide confidence intervals for our estimates highlight the need to examine associations in a larger sample, particularly for maternal hypertension.

A major strength of this study is its prospective longitudinal design as well as the consideration of an “at-risk” sample at baseline. This study addresses a gap in the literature, that of examining several potential determinants for new onset NWMU in children, including adiposity, lifestyle habits, and family history of cardiometabolic disease. Limitations should however be noted. First, the study sample was small (*n* = 193) and inadequately powered to detect smaller effects or to determine the independent effects of multiple determinants on the risk of NWMU. Second, the NWMU phenotype was defined as having at least one cardiometabolic risk factor according to the cutoffs proposed by Cook et al. [14]. There is no consensus on the ideal definition of cardiometabolic risk factors and the use of other definitions may have yielded different results. Moreover, in this sample of young children, the vast majority of participants with an incident NWMU phenotype developed only one cardiometabolic risk factor as opposed to a clustering of cardiometabolic risk factors [3]. In a previous study we have observed that even having a single cardiometabolic risk factor based on a number of definitions among otherwise normal weight children is associated with lower insulin sensitivity 2 years later [15]. We recognize that the definition used in our study may not have captured the full spectrum of the NWMU phenotype. In addition, the short follow-up time of 2 years may have been insufficient for cardiometabolic risk factors to develop and hence to detect associations of interest. Lastly, the generalizability of our findings is restricted to normal weight Caucasian children who have at least one parent with obesity. Replication of our findings in samples of youth from diverse sociodemographic groups is warranted.

Increases in both total body and abdominal adiposity from childhood to early adolescence were identified as determinants for new onset NWMU. Findings highlights the importance of monitoring BMI and waist circumference, even among normal weight children. Future long-term follow-up studies are needed to assess the effects and stability of our findings over time, and to build evidence on whether children should be screened for the presence of metabolic risk factors if they present an increase in weight status even though they remain normal weight per BMI definitions. This study further points to a possible protective effect of physical activity and intake of vegetables and fruits to prevent the development of metabolic complications among normal weight children, although these findings should be confirmed in a larger study.
